# Eating Behaviour among Disabled Athletes in Malaysia

**DOI:** 10.21315/mjms2019.26.3.11

**Published:** 2019-06-28

**Authors:** Haidzir Manaf, Norazmir Md Nor, Nurul Athirah Mohd Azhari, Nur Rabiatul Adawiah Ismut

**Affiliations:** 1 Centre of Physiotherapy, Faculty of Health Sciences, Universiti Teknologi MARA (UiTM) Selangor Branch, Puncak Alam Campus, Bandar Puncak Alam, Selangor, Malaysia; 2 Centre of Nutrition and Dietetics, Faculty of Health Sciences, Universiti Teknologi MARA (UiTM) Selangor Branch, Puncak Alam Campus, Bandar Puncak Alam, Selangor, Malaysia; 3 Clinical & Rehabilitation Exercise Research Group, Faculty of Health Sciences, Universiti Teknologi MARA (UiTM) Selangor Branch, Puncak Alam Campus, Bandar Puncak Alam, Selangor, Malaysia

**Keywords:** disabled athletes, eating behaviour, emotional eating, Malaysia

## Abstract

**Background:**

Nutrition has always been associated with eating behaviour. The eating behaviour can impact not only the normal population but also athletes’ population including disabled athletes too. Athletes have a higher tendency to unhealthy eating behaviour due to intense pressure and competitive environment in sports. It is important to identify the types of eating behaviour among disabled athletes to prevent eating disorder that could affect their performance. Thus, this study aims to identify the types of eating behaviour among disabled athletes in Malaysia.

**Methods:**

This study involved 93 disabled athletes in Malaysia. The setting of the study is at National Sports Council, Malaysia. The data obtained are analysed using chi-square test using SPSS.

**Results:**

This study shows that the most common types of eating behaviour among disabled athletes are emotional eating (37.6%), followed by uncontrolled eating (34.3%) and cognitive restraint (28%). Most of the overweight and obese disabled athletes are prone to emotional eating (19.4%) as compared to non-overweight athletes (18.3%).

**Conclusion:**

As a conclusion, recognising the eating behaviour in disabled athletes is important as more effective and innovative interventions and measures can be undertaken to prevent eating disorder which can enhance sports performance.

## Introduction

Nutrition has always been associated with eating behaviour, which is defined as a complex interaction of physiologic, psychological, social and genetic aspects that effect time of the meal, food consumption quantity, food preference, and selection of food (1). Eating behaviour has become an important factor in assessing the nutritional status of an individual especially on body weight and body mass index (BMI) status. Abnormal attitudes related to maintaining and changing an individual’s body weight are linked to unhealthy eating behaviour (2). Continuous unhealthy eating behaviour such as using diuretics or laxatives to lose weight, self-inflicted vomiting, pathological restriction of food and using anabolic steroids to increase lean mass can lead to psychiatric syndromes like eating disorders (2). Not only that, negative eating behaviour can cause obesity or overweight (1, 3).

The eating behaviour can impact not only normal population but also athletes’ population including disabled athletes. Compared to normal population, athletes have higher frequencies of unhealthy eating behaviour as shown by studies (2). The prevalence of eating disorder in sports are common particularly in weight-sensitive sports such as endurance sports, aesthetic, weight category sports and even jumping events (4). Participation in sports and exercise have shown strong experimental evidence in increasing eating disorder symptomatology (5), in in aesthetic, weight class, or endurance sports, and eating disorder symptoms and dieting are more frequent especially at the elite level (6). Not only these symptoms are common, but it could lead to impair performances and important physical and psychological illnesses too (4). These events occurred as the athletes’ eager to enhance performance and body weight due to them feeling pressured. Several athletes have higher tendency to practice unhealthy methods in changing their body weight or mass of a lean body as they believe that this could improve peak performance. Hence, to prevent eating disorders in athletes from continuing, their eating behaviour must be identified first.

However, researches on eating behaviour in disabled athletes are still uncommon especially in Malaysia although the participation of disabled athletes in sports keeps increasing over year. This increasing number can be proven in the participation of athletes in Paralympic Games over year. According to the International Paralympic Committee (IPC), the number of athletes who participated in Paralympic Games 2014 was 4328 and 4237 in 2012. Thus, this research aims to identify the types of eating behaviour of disabled athletes in Malaysia to provide insight on eating behaviour of disabled athletes in Malaysia.

## Materials and Methods

This study is cross-sectional and quantitative. The total sample size for this study is ninety-three disabled athletes, of 57 males and 36 females. It used purposive sampling, set at the National Sports Council Malaysia. The tool used in this study was a questionnaire named ‘Three Factors Eating Questionnaire-R21’, also known as TFEQ-R21, adapted from Stunkard and Messick, consisting of 51 questionnaire items (7), and validated and tested with Bentler’s Comparative Fit Index (CFI) value of 0.8887 (8). Besides that, TFEQ-R21 has strong association with the original TFEQ through correlation test (9). The TFEQ-R21 consisted of three eating behaviour domains which were cognitive restraint scale, emotional eating scale and the uncontrolled eating scale (9).

To measure BMI status, the weight and height were measured by the researchers. All participants needed to give their consent before participating in this research. The data obtained were analysed using chi-square test through Statistical Package for The Social Sciences (SPSS) version 21. The analysed data is shown in the results.

## Results

The baseline characteristics of the subjects have been identified. Table 1 shows the demographic data of the disabled athletes. Chisquare test was used to determine the differences between male and female of all the independent variables of demographic data which includes age, ethnic, status, smoking, educational, monthly income, working status and BMI. The significant value of each characteristic was set at *P* < 0.05.

Based on Table 1, most disabled athletes are male, which was 56 of the sample size. Based on ethnicity, the highest percentage in this sample was Malay, followed with Chinese, Indian and others with the percentage of 65.6%, 15.1%, 11.8% and 7.5%, respectively. Moreover, the highest percentage in educational level was 64.5% which is a secondary school for both genders, while the lowest percentage was 2.2%, which is postgraduate degree. There was no statistical relationship between gender and educational level, (*P*-value = 0.18).

Based on Table 2, there is no significant difference between the age of male and female athletes. For BMI, there is a significant difference between BMI and gender in which the result for males is M = 25.59 with SD = 5.76 while results for females is M = 22.27 and SD = 6.14. The result shows that disabled male athletes have higher BMI as compared to disabled female athletes.

Based on Table 3, the highest domain type of eating behaviour was emotional eating with the percentage of 37.6%, followed by uncontrolled eating and cognitive restraint with the percentage of 34.4% and 28%, respectively.

From the Table 4, most disabled athletes are non-overweight with the total percentage of 53.8%. Most of the non-overweight disabled athletes are engaged with uncontrolled eating (19.4%) followed by emotional eating and cognitive restriction with the percentage of 18.3% and 16.1%, respectively. For overweight and obese disabled athletes, emotional eating has the highest percentage which is 19.4%, followed by uncontrolled eating and cognitive restriction with 15.1% and 11%, respectively. There was a significant association between BMI and type of eating behaviour with *P*-value = 0.73.

## Discussion

Based on the results, it has been found that disabled female athletes have the lowest BMI mean compared to disabled male athletes, as female athletes are more self-conscious about their body image compared to male athletes. Overall, female athletes tend to focus on body image (10). This is proven in a study focused on intellectually impaired athletes, in which 51% of the disabled female athletes desired a thinner physique as compared to only 37% of male athletes wanted a thinner physique (11). Research in body image in females is more common than in males (12).The body dissatisfaction in women can be due to societal perceptive that thin women are more feminine and attractive (10). Furthermore, research has shown that even in underweight and normal BMI individuals, dissatisfaction about body image is related to weight-related concerns and unhealthy weight loss practice such as crash dieting and vomiting which leads to various health problems including eating disorders and depression (10).

Secondly, this study has found that the most common types of eating behaviour among disabled athletes are emotional eating. Emotional eating can be described as excessive eating in response to negative distress, without specificity to particular moods or emotions (13). In a sub-sample of the obese and overweight samples, a higher quantity of various emotional eating is contributed by perceived stress than in overall sample (13). The emotional eating can be attributed to stress in athletes as stress is common in sports. Disabled athletes that participate in top-level sports are demanded to perform well in pressured circumstances (14). Athletes with disabilities that participate in Paralympic Games can experience intense stress, and it does not limit only to the events itself (15). Not only that, a study has shown that wheelchair basketball players have identified a necessity for consultation in sports psychology so that they can perform better under stress (14).

Thirdly, disabled athletes who engaged in emotional eating types of eating behaviour are mostly from overweight and obese disabled athletes. This has been supported by Psychosomatic Theory of Obesity, a major theory related to emotional eating (13), which theorises that to cope during times of distress, foods are used as an emotional resistance which, sequentially, leads to obesity (16). It further states that obese persons engage in excessive eating in response to negative emotions, while individuals with normal weight have more adaptive coping mechanisms and do not eat in response to emotional distress (13, 17). Thus, emotional eating is connected with the BMI status of an individual including disabled athletes. Meanwhile, most of the non-overweight disabled athletes are engaged in uncontrolled eating behaviour, as the athletes have vigorous training programme and are considered as elite athletes as they are under National Sports Council (MSN) although uncontrolled eating behaviour is often associated with the overweight and obese individuals. Furthermore, uncontrolled eating often leads to binge-eating disorder (BED). Eventhough BED is knowingly linked with obesity, it can also pose a risk to individuals experiencing an intensified external or inner pressure for weight loss (18). Thus, athletes are one of the high risk group for BED as they must adhere to strict requirements for weight and body composition (18). This shows that uncontrolled eating behaviour can occur in non-overweight population especially in athletes’ population due to the stress and adherent to the requirement of their sports.

## Conclusion

In conclusion, this study was designed to determine the types of eating behaviour among disabled athletes in Malaysia. Through this study, it has been identified that the most common types of eating behaviour are emotional eating. However, more research needs to be done targeting disabled athletes due to the lack of research for disabled athletes in Malaysia especially on eating behaviour such as factors influencing eating behaviour so that effective intervention and measure can be undertaken to prevent eating disorder among disabled athletes in Malaysia.

## Figures and Tables

**Table 1:** Demographic data among the disabled athletes

Demographic Data	Male *N* (%)	Female *N* (%)	Total *N* (%)	df	Pearson chi-square value	*P*-value
**Ethnic**				3	1.387	0.24
Malay	39 (41.9)	22 (23.3)	61 (65.6)			
Chinese	10 (10.8)	4 (4.3)	14 (15.1)			
Indian	6 (6.5)	5 (5.4)	11 (11.8)			
Others	2 ( 2.2)	5 (5.4)	7 (7.5)			
Status				1	1.309	0.25
Single	38 (40.9)	28 (30.1)	66 (71.0)			
Married	19 (20.4)	8 (8.6)	27 (29.0)			
Smoking				1	6.205	< 0.01
Yes	18 (19.4)	0 (0.0)	18 (19.4)			
No	39 (41.9)	36 (38.7)	75 (80.6)			
**Educational**				4	16.741	0.18
Primary school	5 (5.4)	3 (3.2)	8 (8.6)			
Secondary school	41 (44.1)	19 (20.4)	60 (64.5)			
Diploma	7 (7.5)	8 (8.6)	15 (16.1)			
Degree	4 (4.3)	4 (4.3)	8 (8.6)			
Postgraduate degree	0 (0.0)	2 (2.2)	2 (2.2)			
Income				3	2.815	0.59
< 1000	14 (15.1)	12 (12.9)	26 (28.0)			
1000–3000	14 (15.1)	8 (8.6)	22 (23.7)			
3000	5 (5.4)	1 (1.1)	6 (6.5)			
Others	24 (25.8)	15 (16.1)	39 (41.9)			
**Working status**				4	16.933	0.06
Employed-full time	30 (32.3)	18 (19.4)	48 (51.6)			
Employed-part time	7 (7.5)	7 (7.5)	14 (15.1)			
Unemployed	19 (20.4)	6 (6.5)	25 (26.9)			
Retired	1 (1.1)	2 (2.2)	3 (3.2)			
Student	0 (0.0)	3 (3.2)	3 (3.2)			

**Table 2:** Age and BMI of the disabled athletes

	Male Mean (SD)	Female Mean (SD)	*P*-value
Age	31.56 (10.66)	31.92 (11.63)	0.87
BMI	25.59 (5.76)	22.27 (6.14)	0.01

**Table 3:** Types of eating behaviour

Type of eating behaviour	*N* (%)	Mean (SD)
Cognitive Restraint (CR)	26 (28.0)	2.09 (0.80)
Uncontrolled Eating (UE)	32 (34.4)	
Emotional Eating (EE)	35 (37.6)	

**Table 4:** Comparison of types of eating behaviour and BMI groups among the disabled athletes

Variables (BMI groups)	Domain			Total [*N* (%)]	df	Pearson chi-square test value	*P*-value

	CR [*N* (%)]	UE [*N* (%)]	EE [*N* (%)]				
Non-overweight (< 25 kg/m^2^)	15(16.1)	18(19.4)	17(18.3)	50(53.8)	2	0.621	0.73
Overweight and obese (≥ 25 kg/m^2^)	11(11.8)	14(15.1)	18(19.4)	43(46.2)			
